# Why alternative memory and place-making practices in divided cities matter

**DOI:** 10.1080/13562576.2019.1637251

**Published:** 2019-07-14

**Authors:** Monika Palmberger

**Affiliations:** a Department of Social and Cultural Anthropology, University of Vienna, Vienna, Austria; b Interculturalism, Migration and Minorities Research Centre, University of Leuven, Leuven, Belgium

**Keywords:** Memory, place-making, generation, Bosnia and Herzegovina, divided city, nostalgia

The clear aim of this special issue is to move beyond ethno-national divisions and to show how Mostar – like other ‘divided’ cities – is more than its conflict, nationalism and ultimately its division. However, this confronts us with a paradox: how do we push research beyond ethno-national divisions while simultaneously acknowledging that those same divisions are the starting point for the contributors’ analysis? In this intervention piece I offer one possible way to address this paradox by focusing on practices of place-making and drawing on the repositioning of memories in the city. This allows me to elaborate on a specific focus offered by the papers in this special issue encountered in practices of place-making, sense-making and memory-making. By taking this angle I wish to explore the particularities of the Mostar case but at the same time to go beyond it and tackle issues that are likely to affect other cities sharing a similar fate. In order to do so, I will build on the articles’ findings as well as on my own findings from my fieldwork in Mostar from 2005 until 2008, followed by several revisits.

## Memory, place-making and the specific role of nostalgia

Most scholarly and media discussions on memory in the Yugoslav successor states centre on what we may call ‘public memory’, as is actively propagated by politicians, historians and journalists, among others. However, this debate has offered little insight into the ways in which individuals – in our case, Mostarians – position themselves relative to the past. Moreover, it too easily paints a picture of memory politics as a top-down process whereby citizens are depicted as empty containers that passively accept these politics wholesale.

One central insight I gained during my fieldwork in Mostar was that Mostarians are not only exposed to changing political contexts but are also confronted with their personal past experiences; therefore their reconstructions of the past remain more flexible and situational than those of people professionally involved in writing official national histories. While the latter present a goal-oriented narrative, the reconstructions of the former can be better described as target-seeking (Palmberger, 2016). Here I adapt Michel de Certeau’s distinction between strategy and tactic. Discursive strategies, as I understand them, are employed by those who claim to represent the nation in order to narrate independent, coherent national histories, to legitimize and objectify them. A tactic, in de Certeau’s sense, is utilized by individuals to create space for themselves in a field of power (de Certeau, 1980). Let me briefly exemplify this in the process of renaming the cityscape in Mostar.

In Mostar, as in many other places in Bosnia and Herzegovina and other Yugoslav successor states, the cityscape has been nationalized by means of renaming streets, squares and other public places. This act of renaming (particularly by Croat nationalist elites) has literally inscribed ethno-national divisions onto the city of Mostar. Still, it would be wrong to assume a direct link between a national historiography inscribed in the cityscape by cultural, academic and political elites and how people interpret the ethno-national markers they encounter in everyday life. In the case of street names – as for memorials and commemorative sites more generally – this implies that people need to read them, which means they first have to notice and pay attention to them. During my fieldwork, I observed that Mostarians were often unaware of the new street names and other urban toponyms and continued to refer to them by their old names (Palmberger, 2018).

The renaming of streets has not only been ignored: sometimes it has also been outright resisted. This was the case, for example, in Sarajevo, when citizens actively protested against renaming its main artery, ulica Maršala Tita (named after the Yugoslav statesman Josip Broz Tito), in honour of Alija Izetbegović (a Bosniak activist and the first president of Bosnia and Herzegovina). Here it became evident that the decisions of the cultural, academic and political elites about what should be publicly remembered and what should be silenced did not resonate with the views of a good part of Sarajevo’s citizens (Palmberger, 2018; Robinson, Engelstoft, & Pobric, 2001). People took to the streets in protest because they did not want to erase the memory of their former president. This is one way in which people attach alternative meanings to places, similar to what Forde in this special issue calls ‘rescripting’ of space. Rescripting of space is tightly entangled with rescripting relations and it shows ‘the transformative capabilities of the social use of space’ (Forde, this volume). Forde identifies these processes of rescripting particularly among youth, who, for example, question, subvert and transform mono-ethnic school spaces. In a similar way, in this issue Laketa analyzes how youth negotiate heterogeneous and complex landscapes of division. One such negotiation described by Laveta was an event called ‘Bridging the Divide’ that took place in 2013. During this march, approximately 300 students converged on the Old Bridge from East and West Mostar. By marching from both sides of the city and meeting at this historic site, students transformed the bridge from a place of division to one of togetherness. Despite the relatively high number of participants, the event did not make it into the local news, most likely because it did not fit into the dominant narrative of division and conflict.

### Places of alternative remembrance

Several contributors to this special issue identify spaces of alternative/oppositional remembrance based on a shared Yugoslav past. Nostalgic discourses of Tito’s Yugoslavia persist and vividly so in Mostar’s case, not only among Bosniaks but also Croats. In the following paragraphs I will elaborate on what this approach to memory – going beyond the scope of memory politics – means and what we can gain from it.

Memory, specifically nostalgic memories of pre-war/socialist Yugoslavia and the place-making practices they are entangled with are central in this special issue. Several of the articles describe the multiple entanglements between past, present and future in contemporary Mostar and specifically how the past (and pre-war/socialist memories thereof) can be understood as an orienting force for the future. Central here are place-making practices that create alternative spaces where an imagined (and to some extent also glorified) shared Yugoslav past is nurtured. These memory- and place-making practices go beyond ethno-national divisions to envision a different future. Such place-making practices can be subtle attempts to rescript Mostar’s landscape, as described by Laketa and Forde in the case of Mostar’s youth. But they may also result in the making of places dedicated to remembering a shared past and thereby providing room for a shared future, as described by Wollentz and colleagues in the case of heritage making and the workers’ monument, or the opening of Yugo-nostalgic cafes as described by Summa.

Wollentz and colleagues draw attention to a shared past that has had no public representation in post-war and post-socialist Mostar: that of workers. They analyze in detail a grassroots initiative to build an alternative monument for the Rudnik coal mine workers. By examining the making of this alternative monument which commemorates the shared identity of Yugoslavs in general and Yugoslav workers in particular, the authors emphasize nostalgia’s progressive potential and how it can act as the ‘basis of action’ across generations. What we encounter in the specific case of this memorial is interesting in so far as the initiative came mainly from young people, who themselves have no memory or limited memories of socialist Yugoslavia and who approached the former workers with the aim of creating a shared monument. The motivation to build this alternative monument can be interpreted as a tactic to create alternative spaces in a city that is determined by ethno-national remembrance. Moreover, the monument represents not only an alternative place of remembrance but also a participatory one. Rather than implementing a history from above, the initiators aimed to create this monument together with the neighbourhood community, including people of different generations. Abrašević, an alternative youth centre in Mostar, represents, as Carabelli (2018) shows, a similar place, where young and old can meet and are invited to explore the past, particularly those aspects of it that can be used creatively in the present. Thereby it offers the space needed to ‘critically engage with nostalgia as a reflective tool’ (Carabelli, 2018, p. 131).

Mostar’s café Boemi, which Summa vividly describes in her contribution to this special issue, is another example of how memories of the Yugoslav past have a strong political meaning in present-day Mostar, often in opposition to current politics and also ways of life. Different to the Rudnik mine monument – Café Boemi is more homogenous in terms of generation and gender. Here elderly men belonging to the pre-war ‘elite’ cherish pre-war times. Analyzing the encounters in this café, Summa makes clear that what binds its customers together is not only their anti-nationalist ideas about a divided city but also their particular identity and pride in being ‘true’ Mostarians. This opens up a discussion of other demarcation lines present in Mostar, which are often overlooked. In the case of Café Boemi, Summa identifies three additional lines of division that go beyond the ethno-national one: the division between politicians and ordinary people; between true/‘native’ Mostarians (who mainly belong to the pre-war urban elite) and newcomers; and finally the division between those who cross sides in Mostar and those who do not. The last two categories are particularly important for customers at Boemi café, who strongly identify with the pre-war urban elite and with those who cross borders (in contrast to those who do not). By identifying with the pre-war urban elite and with those open-minded enough to cross borders and who apparently do not categorize people according to their ethno-national background, they claim some form of superiority. These (alternative) lines of division have already been identified by scholars who did research in Bosnia and Herzegovina in the years immediately following the war (see, for example, Jansen, 2005; Stefansson, 2010). However, as this special issue shows, these lines of division remain. Readers may ask themselves what we gain by opening up more lines of division in Mostar. One answer contributors to this special issue provide is to show complexity, to which I would add another: to show commonalities, shared pasts and presents that transcend ethno-national lines, as I will elaborate below in relation to ‘border crossing’ practices.

The articles in this special issue not only complexify lines of divisions in Mostar but they also question common understandings of these lines of division. In this respect, Djurasovic’s contribution questions the highly prioritized aim (mostly promoted by actors of the so-called ‘international community’) to reconcile ethno-national divisions. Together with her interviewees Djurasovic suggests that divisions can be found in all cities and that it is sometimes more important to acknowledge them than to immediately seek a remedy to overcome them. Moreover, many of her interviewees made clear that other issues are more pressing for them, such as the economic crisis and the dire job market. Rather than putting all the emphasis on ‘reconciliation’ (often without laying open what reconciliation indeed requires and means), the authors in this special issue instead suggest taking into account other lines of division at the same time as observing and analyzing not only how the different forms of division are (re-)made but also how they are questioned, countered and overcome in everyday spatial practices and interactions. In Summa’s words ‘The focus on the everyday, therefore, allows us to understand the city as a place in movement and built through multiple meanings – drifting away from the rigid cartographic representation of the divided city’ (Summa, this volume).

The re-enactment of pre-war memories and the creation of shared places that this special issue reveals contribute to the disruption of the ‘divided city’ narrative. This resonates very well with my work in which I have shown how positive memories of pre-war cross-national relations have a strong integrative potential and how practices of post-war ‘border crossing’, as I call it, draw heavily on these memories (Palmberger, 2013b). These memories around an idealized past can become powerful tales put forward in support of a shared future as well as in concrete cross-border interactions to establish common ground. Thereby ‘border crossing’ is understood not only as a physical act of crossing sides (as in Mostar between the Croat-dominated west and the Bosniak-dominated east side) but also in a more metonymical sense, for example, when absolute and exclusive national identities are questioned. Border crossing thus implies acts of scrutinizing and deconstructing national identities as well as the reconquest of the city and the reintegration of ‘the other side’ into one’s everyday life (Palmberger, 2013b). Or in the words of Summa ‘spatial practices are not only expressed through movement (crossing the Bulevar or not, for example), but also through disputing and reshifting meanings of places and by establishing new meeting places to resist a drive to homogenization and segregation’ (Summa, this volume).

### Youth, the role of generation and the transmission of memories

I would like to end this intervention piece with a note on generations and the particular role ascribed to youth in post-war and divided societies. Generation can provide an important analytical focus in such contexts, since different generations share often very distinct personal experiences (past and present). Furthermore, generation is tightly coupled with questions of transmission (of traumatic memories) and, more broadly, the possibility of change (Palmberger, 2016). Recent literature, in particular on the Yugoslav context, shows that we cannot assume the direct transmission of (traumatic) memories down the generations (see Palmberger, 2010). Sorabji (2006), for example, in her article, ‘Managing Memories in Post-War Sarajevo’ shows how transferred memories are scrutinized, contextualized and selectively adopted to accommodate personal worldviews in deciding what and what not to pass on. It is this field of tension between the collective and the personal, and between persistence and change that comes to the fore in what I call ‘generational positioning’ (Palmberger, 2016).

While not an explicit focus, the question of generations – particularly the younger generation – runs through most of the contributions to this special issue. The contributors seem particularly concerned with young people’s perceptions and life prospects. Laketa points out ‘there has been a tendency to accentuate the political, and indeed to demonstrate children and youth’s revolutionary potential as subjects for resistance and agents of subversion’ (Laketa, this volume). This is also the case in Mostar. Interestingly, however, at the same time youth in Mostar are stigmatized as the generation that grew up under ethno-national divisions and are said not to know anything different (often this situation is also characterized by a lack of pre-war memories). Judging from my own fieldwork and from the detailed descriptions this special issue offers, there is potential among local youth for both. On one hand, there is the potential to take ethno-national lines of divisions as unquestioned, perceiving them as ‘natural’ rather than ‘created’. Here the ‘segregated educational landscapes work forcefully to entrench fixed notions of identity’ (Laketa, this volume). On the other hand, youth in Mostar actively engage in border crossing, as the contributions in this special issue vividly show. Even if pre-war memories are limited, young people too draw on the pre-war/Yugoslav past as a ‘compass’ for the future and for working against divisions. Thus, nostalgia for Yugoslavia is not only reserved for those who have vivid personal memories of this period but the younger generation can also skillfully appropriate nostalgia for Yugoslavia (Palmberger, 2008).

Regardless of generational background, what these nostalgic memories of a shared Yugoslav past have in common is that they counter the ‘illusion of a natural and fixed ethnic urban division’ (Laketa, this volume). These individual memories of positive pre-war coexistence, however, are politically marginalized in public discourse in order to enforce the nationalist politics of official histories that centre on national suppression and collective victimhood (see Palmberger, 2006). Interestingly, however, even proponents of this nationalist discourse, who primarily stress national suppression under Tito, often simultaneously engage in a parallel discourse of good-neighbourliness and a fulfilled life during this particular period (Palmberger, 2013a). Of course, not everyone preserving positive memories of the national coexistence during Yugoslav times is keen to re-establish cross-national relationships. Nevertheless, emphasizing – also in scholarly work – what works and what has worked in the past is likely to have a positive and trust-building effect (Dembinska, 2010). The aim of the present special issue as formulated by the editors is exactly this: to ‘encourage conversations among scholars of “divided cities” and to further this conversation by asking what would it change if we knew more about how these cities challenge (rather than reinforce) their ethnic divisions’ (Carabelli et al, 2019). This change of scholarly focus is likely not only to affect academic debates but also to help build confidence and trust in said cities.

## References

[CIT0001] Carabelli, G. (2018). *The divided city and the grassroots. The (un)making of ethnic divisions in Mostar*. London: Palgrave Macmillan.

[CIT0001a] Carabelli, G., Djurasovic, A., & Summa, R. (2019). Challenging the representation of ethnically divided cities: Perspectives from Mostar. *Space and Polity*. doi:10.1080/13562576.2019.1634467.

[CIT0002] de Certeau, M., Jameson, F., & Lovitt, C. (1980). On the oppositional practices of everyday life. *Social Text*, 3, 3–43. doi: 10.2307/466341

[CIT0003] Dembinska, M. (2010). Building trust: Managing common past and symbolic public spaces in divided societies. *Ethnopolitics*, 9(3), 311–332. doi: 10.1080/17449050903564845

[CIT0004] Jansen, S. (2005). Who’s afraid of white socks? Towards a critical understanding of post-yugoslav urban self-perceptions. *Ethnologia Balkanica*, 9, 151–167.

[CIT0005] Palmberger, M. (2006). Making and breaking boundaries: Memory discourses and memory politics in Bosnia and Herzegovina. In M. Bufon (Ed.), *The Western Balkans – a European challenge. On the decennial of the Dayton Peace Agreement* (pp. 525–536). Koper: Založba Annales.

[CIT0006] Palmberger, M. (2008). Nostalgia matters: Nostalgia for Yugoslavia as potential vision for a better future? Sociologija. *Casopis za sociologiju, socijalnu psihologiju i socijalnu antropologiju*, 50(4), 355–370.

[CIT0007] Palmberger, M. (2010). Distancing personal experiences from the collective: Discursive tactics among youth in post-war Mostar. *L’Europe en formation: Journal of Studies on European Integration and Federalism*, 357, 107–124. doi: 10.3917/eufor.357.0107

[CIT0008] Palmberger, M. (2013a). Ruptured pasts and captured futures: Life narratives in post-war Mostar. *Focaal – Journal of Global and Historical Anthropology*, 66, 14–24.

[CIT0009] Palmberger, M. (2013b). Acts of border crossing in post-war Bosnia and Herzegovina: The case of Mostar. *Identities: Global Studies in Culture and Power*, 20(5), 544–560. doi: 10.1080/1070289X.2013.822799

[CIT0010] Palmberger, M. (2016). *How generations remember: Conflicting histories and shared memories in post-war Bosnia and Herzegovina*. London: Palgrave Macmillan.

[CIT0011] Palmberger, M. (2018). Renaming streets and nationalizing public space: The case of Mostar, Bosnia and Herzegovina. In R. Rose-Redwood, D. Alderman, & M. Azaryahu (Eds.), *The political life of urban streetscapes: Naming, politics, and place* (pp. 168–184). London: Routledge.

[CIT0012] Robinson, G., Engelstoft, S., & Pobric, A. (2001). Remaking Sarajevo: Bosnian nationalism after the Dayton accord. *Political Geography*, 20(8), 957–980. doi: 10.1016/S0962-6298(01)00040-3

[CIT0013] Sorabji, C. (2006). Managing memories in post-war Sarajevo: Individuals, bad memories, and new wars. *Journal of the Royal Anthropological Institute*, 12(1), 1–18. doi: 10.1111/j.1467-9655.2006.00278.x

[CIT0014] Stefansson, A. (2010). Coffee after cleansing? Co-existence, co-operation, and communication in post-conflict Bosnia and Herzegovina. *Focaal – Journal of Global and Historical Anthropology*, 57, 62–76.

